# Quinone Reductase 2 Dimerization is Dynamically Driven by Ligand Binding

**DOI:** 10.1021/acs.biochem.6c00461

**Published:** 2026-09-24

**Authors:** Maya Miller, Dan Loewenthal, Philipp Kukura, Nathaniel Gould

**Affiliations:** † Department of Physiology, Anatomy and Genetics, 6396 University of Oxford, Oxford OX1 3PT, U.K.; ‡ Department of Chemistry, University of Oxford, Oxford OX1 3TA, U.K.; § Kavli Institute for Nanoscience Discovery, University of Oxford, Oxford OX1 3QU, U.K.; ∥ Department of Psychiatry, University of Oxford, Oxford OX3 7JX, U.K.

## Abstract

Human quinone reductase 2 is a cytosolic flavoprotein involved in cell physiology and metabolism, and implicated in several diseases. However, the mechanisms that govern its oligomeric assembly and diverse functional outcomes remain incompletely understood. Here, we employ native mass spectrometry to directly resolve the dynamic oligomeric landscape of recombinant human quinone reductase 2 expressed in *Escherichia coli*, preserving noncovalent interactions and enabling analysis of assembly behavior under native conditions. Quinone reductase 2 is predominantly observed as a dimer stabilized by multiple noncovalently bound ligands, giving rise to discrete species. Top-down native mass spectrometry reveals a single intact proteoform, excluding covalent modification or covalently bound flavins as drivers of oligomerization. Binding of flavin adenine dinucleotide robustly stabilizes the dimer, while unexpectedly, flavin mononucleotide also promotes dimer formation. As flavin mononucleotide and flavin adenine dinucleotide differ structurally by the presence of an adenine dinucleotide moiety, we hypothesized that purine nucleotide binding itself may modulate quinone reductase 2 assembly. Consistent with this, we identify a new concentration-dependent effect of guanosine triphosphate on quinone reductase 2 dimerization. Functional reductase assays show that flavin-stabilized dimers exhibit the highest catalytic activity, whereas guanosine triphosphate-induced dimers retain reduced activity. Binding of the inhibitor YB537 abolishes activity despite promoting dimer formation. Together, these findings reveal ligand-dependent structural plasticity in quinone reductase 2 oligomerization that is decoupled from reductase activity. This supports a model in which dimerization is shaped by the local ligand environment, enabling distinct functional states and suggesting a broader regulatory role beyond reductase catalysis.

## Graphical abstract

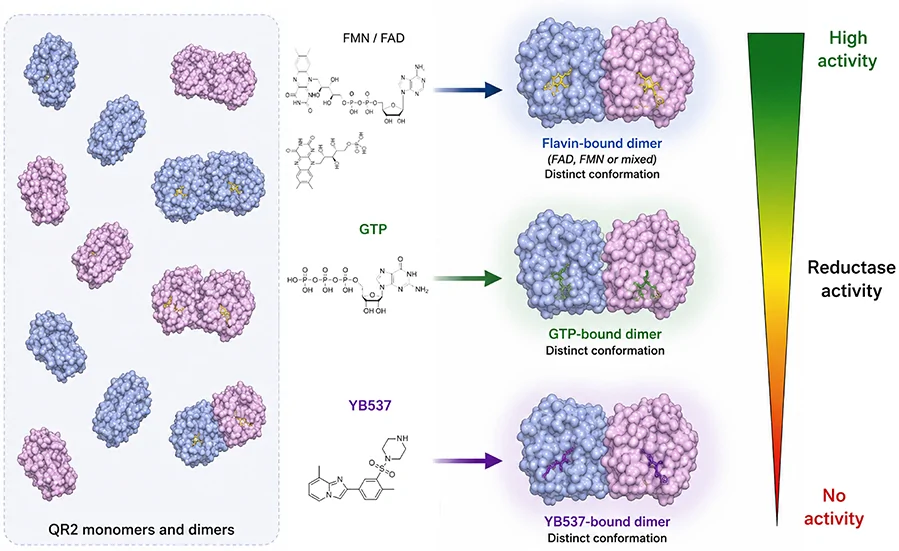

## 1 Introduction

Quinone reductases play a central role in cellular redox homeostasis by catalyzing the reduction of quinones and related substrates, thus limiting the generation of reactive oxygen species and contributing to detoxification pathways.^1^ In humans, quinone reductase 2 (QR2), also known as NQO2, is a cytosolic flavoprotein that shares significant sequence and structural similarity with quinone reductase 1 (QR1/NQO1), but exhibits distinct biochemical properties and biological functions.^2,3^ QR1 is well characterized as a NAD­(P)­H-dependent detoxifying enzyme with broadly defined cytoprotective functions, while phylogenetic and ancestral reconstruction analyses show QR2 diverged under selective pressure away from QR1 and the use of NAD­(P)­H.^4,5^ QR2 is broadly conserved across vertebrates,^5–7^ is widely expressed in variable levels across tissues,^8^ and has been linked to a range of context-dependent functions, including metabolic regulation and neuronal physiology, among others.^9^ Importantly, QR2 is also involved in toxification processes, and its dysregulation is observed in cancer and neurodegenerative diseases.^9,10^ To wit, QR2 inhibitors are neuroprotective and have rescued mice models of Alzheimer’s disease.^11,12^ Despite growing evidence linking QR2 to important physiological roles and disease contexts, the molecular mechanisms underlying QR2 regulation and function are incompletely elucidated.^9^

Crystallographic and biochemical studies have shown that QR2 is a homodimeric enzyme, dependent upon flavin adenine dinucleotide (FAD) association for stability and catalytic competence.^3^ Additional structure–function studies have shown that FAD redox state and local sequence variation can influence QR2 conformation, stability and ligand recognition.^13,14^ Despite these advances, most of the existing structural insights into QR2 are derived from static, high-concentration conditions, providing limited information about the dynamic nature of its oligomeric assembly in solution. Consequently, it remains unclear whether QR2 dimerization is solely driven by protein concentration or is also actively regulated by small molecules. This question is especially pertinent given the many documented interactions QR2 has with a variety of endogenous and exogenous ligands, including monoamines, acetaminophen, and various naturally occurring and pharmacologically derived inhibitors.^2,9,14–18^ However, the impact of these interactions on its oligomeric assembly have not been systematically explored.

Despite substantial sequence similarity between QR1 and QR2, accumulating evidence indicates that these enzymes are regulated through distinct structural and ligand-dependent mechanisms. Mechanism-based inhibition studies have demonstrated that small molecules can selectively target QR2 over QR1 by exploiting differences in the architecture of the active site and the position of FAD, highlighting the fundamental structural divergence between the two enzymes despite their partial homology.^2,12,19,20^ In QR1, ligand binding has been shown to modulate enzymatic activity through conformational communication across the dimer interface, establishing that functional output depends not only on dimerization but also on the specific conformation adopted by the dimeric assembly.^21^ These observations provide a conceptual framework for understanding how ligand-induced remodeling of the QR2 quaternary structure may regulate reductase function.

Native mass spectrometry (nMS) provides a powerful tool to interrogate protein assembly and ligand binding under near physiological conditions.^22,23^ By preserving noncovalent interactions during ionization, nMS enables direct observation of coexisting oligomeric and ligand-bound species, allowing subtle shifts in equilibrium to be resolved without the need for labeling or immobilization.^23,24^ As such, nMS is uniquely suited to dissecting ligand-regulated assembly mechanisms in proteins whose functional states may depend on dynamic oligomerization.

In this study, we applied nMS to investigate the oligomerization behavior of recombinant human QR2 expressed and purified from *Escherichia coli*. We examine how flavin cofactors, nucleotides, and a selective small-molecule inhibitor influence monomer–dimer formation. By integrating nMS with a top-down approach, mass photometry and functional enzymatic assays, we demonstrate that QR2 assembly is sensitive to ligand occupancy, with noncovalent ligand binding shifting the distribution of oligomeric states. This represents a central regulatory mechanism that links the function of QR2 with the cellular chemical environment, particularly in the context of brain physiology and disease.

## 2 Materials and Methods

### 2.1 Protein Expression and Purification

The plasmid encoding human quinone reductase 2 (hNQO2; NM_000904.6) with an N-terminal 6 × His tag followed by a TEV protease cleavage site was obtained in the bacterial expression vector pET-6xHis/TEV (Vector ID: VB240530–1459jna). Plasmids were amplified by transforming into *E. coli* UltraStable cells and confirmed by Sanger sequencing. For protein expression, the verified plasmid was transformed into chemically competent *E. coli* BL21­(DE3) cells. Overnight preculture was grown at 37 °C by inoculating a freshly transformed colony in a 100 mL LB media. The preculture was diluted 1:100 into fresh 6 × 1 L LB media and was grown at 37 °C until the OD600 reached 0.6. Cells were induced by the addition of 0.5 mM isopropyl β-D-1-thiogalactopyranoside (IPTG), and the culture was grown for another 3 h at 37 °C. Cells were pelleted using centrifugation with the Beckman JLA 8.1000 rotor for 15 min at 5000 × g. The pellet was resuspended in lysis buffer consisting of 20 mM Tris-HCl (pH 8.0), 150 mM NaCl, and 10% (v/v) glycerol, supplemented with an EDTA-free protease inhibitor cocktail. The lysed cells were then centrifuged at 4 °C to separate the insoluble material using a JA-25.50 rotor at 20,000 × g, 30 min. The clarified supernatant was collected and subjected to His-tag affinity purification to isolate recombinant hNQO2.

### 2.2 Native and Top-Down Mass Spectrometry

Purified QR2 was buffer-exchanged into 200 mM ammonium acetate (pH 8.0) using gel-filtration spin columns (75 μL Zeba Spin Desalting Columns; Thermo Fisher Scientific) immediately prior to analysis. For ligand-spiking experiments, YB537,^12^ GTP (Sigma-Aldrich, G8877), FAD (Fisher Scientific, 11411838), and FMN (Cambridge Bioscience, 332–10509–2) were individually weighed and dissolved directly in 200 mM ammonium acetate (pH 8.0) to prepare 1 mM stock solutions. Ligands were then diluted to the indicated final concentrations directly into the QR2 samples immediately prior to native mass spectrometric analysis, as specified in the corresponding spectra.

Native mass spectrometry measurements were performed on a Q Exactive UHMR mass spectrometer (Thermo Fisher Scientific) operated in positive ion mode using the manufacturer’s recommended settings for native mass spectrometry. Spectra were acquired at a resolving power of 12,500 (at *m*/*z* 200), with HCD set to 10, in-source trapping (IST) set to 50, and an acquisition range of *m*/*z* 350–10,000. Nanoelectrospray ionization was achieved using in-house prepared gold-coated borosilicate capillaries supplied with a slight backing pressure (approximately 0.5 mbar) and held at 1.0 kV relative to the instrument inlet. The inlet capillary temperature was maintained at 200 °C.

Top-down mass spectrometry was performed on intact QR2 using an Orbitrap Eclipse Tribrid mass spectrometer (Thermo Fisher Scientific) operated in positive ion mode with higher-energy collisional dissociation (HCD). Native protein ions were generated by nanoelectrospray ionization using a spray voltage of 1.0 kV relative to the instrument sampling interface (200 °C), with 200 V in-source activation applied for desolvation. The instrument was operated in intact protein mode under high-pressure conditions. MS1 spectra were acquired over an *m*/*z* range of 500–8,000 at a resolving power of 120,000. MS2 spectra were acquired using HCD with a collision energy of 200 V.

Raw spectra were processed and deconvoluted using PrecisION software.^25^ Observed intact masses were compared with the theoretical sequence mass to assess proteoform heterogeneity using a mass tolerance threshold of ±3 Da.

### 2.3 *In Silico* Modeling

To compare QR2-ligand interactions, the resolved crystal structure of QR2 with FAD (PDB: 7O4D) and FMN (PDB: 4QOF) were loaded to LigPlot^26^ and to PLIP,^27^ to provide a comparative itemization of interactions. Only interactions that were supported by both software packages were referenced in the analysis. No crystal structure exists for ATP/GTP. Therefore, the QR2 sequence from PDB crystal structure 7O4D was used in AlphaFold,^28^ and the predicted structure of QR2 bound to ATP/GTP was then modeled. The resulting model was loaded into ChimeraX, converted into PDB files, and QR2–ATP/GTP ligand interactions were itemized with LigPlot and PLIP similarly to FAD and FMN as described above.

### 2.4 Mass Photometry

Measurements were performed on a TwoMP mass photometer (Refeyn).^29^ Glass coverslips (Epredia no. 1.5 coverslips, 50 × 24 mm, VWR, cat. no. 16002–264) were cleaned by 5 min ultrasonication in 50:50 IPA:MQ (24137–2.5L-M, Sigma-Aldrich), followed by 5 min ultrasonication in Milli-Q (MQ) water. Silicone gaskets (CultureWell, CW-50R-1.0, 50–3 mm diameter × 1 mm depth) were placed on the coverslips. Samples were measured in PBS at a final concentration of 50 nM QR2, in the presence or absence of 1 μM FAD/FMN/GTP. Data were acquired for 60 s at 237 frames s^–1^ using AcquireMP (Refeyn, 2023 R1.1). The resulting movies were analyzed using DiscoverMP (Refeyn, v2024 R1) and *K*_d_ values for monomer–dimer equilibrium were calculated according to a previously published procedure.^30^

### 2.5 Enzymatic Activity

The activity of QR2 reductase was quantified by measuring the oxidation rate of the synthetic cofactor benzyl dihydro-nicotinamide (BNAH), as previously described.^12^ Briefly, a final concentration of 100 μM BNAH (Fluorochem, F545103) and 100 μM menadione (Fluorochem, F049845) were dissolved into a buffer of 50 mM Tris with 150 mM NaCl at pH 8.5, in the presence of 10 nM recombinant human QR2, with increasing concentrations of YB537,^12^ GTP (Sigma-Aldrich, G8877), FAD (Fisher Scientific, 11411838), or FMN (Cambridge Bioscience, 332–10509–2). BNAH oxidation was measured by absorption at 350 nm in a reaction volume of 120 μL with a PHERAstar FSX plate reader (BMG Labtech), using a 96 well clear plate format (Scientific Laboratory Supplies, Corning-3997). The initial velocity was determined and compared across conditions. The activity was measured in triplicates.

## 3 Results and Discussion

### 3.1 Ligand-Associated Oligomerization of Human QR2 Revealed by Native Mass Spectrometry

Recombinant human QR2 was heterologously expressed in *E. coli*, which provides a robust expression platform that shares significant overlap with small molecules found in mammalian cells.^31,32^ QR2 was purified by nickel-affinity chromatography without further sample manipulation or deliberate cofactor supplementation. nMS of the purified protein revealed two well-resolved charge state distributions corresponding to monomeric and dimeric QR2, consistent with their respective molecular masses (Figure 1A,B). In addition to apo form, the dimeric population exhibited pronounced heterogeneity, characterized by multiple discrete mass shifts indicative of small molecule binding.

**Figure 1:**
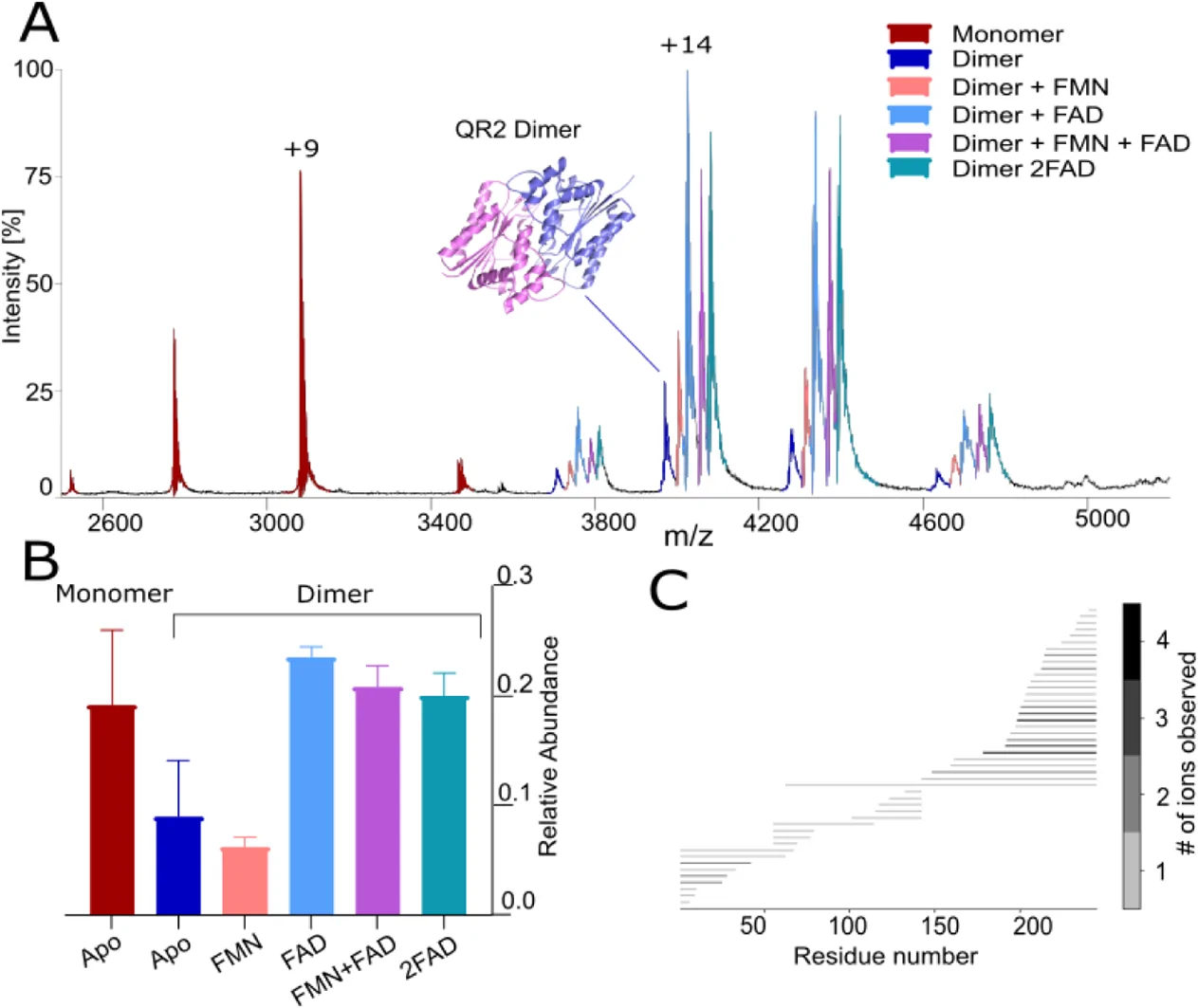
Native mass spectrometry characterization of QR2 and associated ligand-bound states. (A) Native mass spectrum of 10 μM recombinant human QR2 showing coexisting monomeric (red) and dimeric species (color-coded by ligand-binding state). The dimeric region reveals apo dimer (dark blue) and four predominant noncovalent adducts corresponding to binding of a single FMN (salmon), a single FAD (light blue), a mixed FMN-FAD species (purple), and two FAD molecules (cyan) per QR2 dimer. An inset shows the predicted QR2 homodimer structure generated using AlphaFold.^28^ (B) Relative abundance of the individual dimer-associated binding states derived from native mass spectra. (C) Top-down native mass spectrometry of intact QR2 showing fragment ion coverage across the protein sequence, indicating the absence of covalently bound small molecules or post-translational modifications.

nMS showed that at 10 μM, the estimated physiological concentration,^33^ QR2 is predominantly observed as a dimer, indicating that this represents the principal oligomeric form of the protein in solution (Figure 1A,B). Within the dimeric population, four reproducible ligand-associated species were resolved, corresponding to mass increases of +456, +786, +1241, and +1570 Da relative to the apo dimer. Based on mass agreement with known cofactor masses, these species are consistent with dimers bound to a single FMN, a single FAD, both FAD and FMN, or two FAD molecules, respectively. The presence of multiple well-resolved cofactor-associated dimers in the absence of exogenous ligand addition indicates that QR2 purified from *E. coli* exists as an ensemble of ligand-bound states. Intracellular concentrations of FAD and FMN in *E. coli* have previously been reported at approximately 50–200 μM and 3–50 μM, respectively, within a total metabolite pool of ∼300 mM.^34^ Quantitative analysis revealed that the apo dimer represents only a minor fraction of the total dimeric population, whereas flavin-associated dimers dominate, with FAD-containing species forming the most abundant states. Together, these observations suggest that flavin binding strongly stabilizes the QR2 dimer and that the surrounding small-molecule environment shapes both the composition and relative abundance of distinct dimeric assemblies.

To determine whether variation in covalent protein modification contributes to this behavior, intact QR2 was further analyzed by top-down nMS (Figure 1C and Supplementary Figure S1). Spectra acquired on an Orbitrap mass spectrometer using higher-energy collisional dissociation (HCD) revealed a single dominant intact protein form. Deconvolution using PrecisION^25^ yielded a molecular mass consistent with the theoretical sequence mass within ±3 Da. Fragment ion analysis provided 18% sequence coverage, with ions distributed throughout the N-terminus, internal regions, and C-terminus, as confirmed by fragment position and coverage maps (Supplementary Figure S1). No mass shifts or fragmentation patterns consistent with post-translational modifications or covalently bound cofactors were detected at any position along the sequence. These data exclude covalent modification, including rare covalent flavin attachment,^35^ as contributors to QR2 oligomerization.

Collectively, these results demonstrate that recombinant human QR2 exists as a heterogeneous ensemble of noncovalently ligand-associated oligomeric states. The presence of multiple cofactor-bound dimer species, together with the absence of covalent modification, indicates that QR2 assembly is likely to be governed by its chemical microenvironment.

### 3.2 Structural Analysis of Ligand Interactions with QR2

To investigate how ligand binding contributes to QR2 dimerization, we first analyzed flavins associated with the predominant dimeric species. Comparison of the chemical structures of FAD and FMN (Figure 2A) shows that FAD comprises an FMN core linked to an adenine nucleotide moiety, which is absent from FMN. This structural difference raises the possibility that interactions involving the additional adenine nucleotide moiety could contribute to dimer stabilization, motivating investigation of purine nucleotides as potential modulators of QR2 dimer assembly.

**Figure 2:**
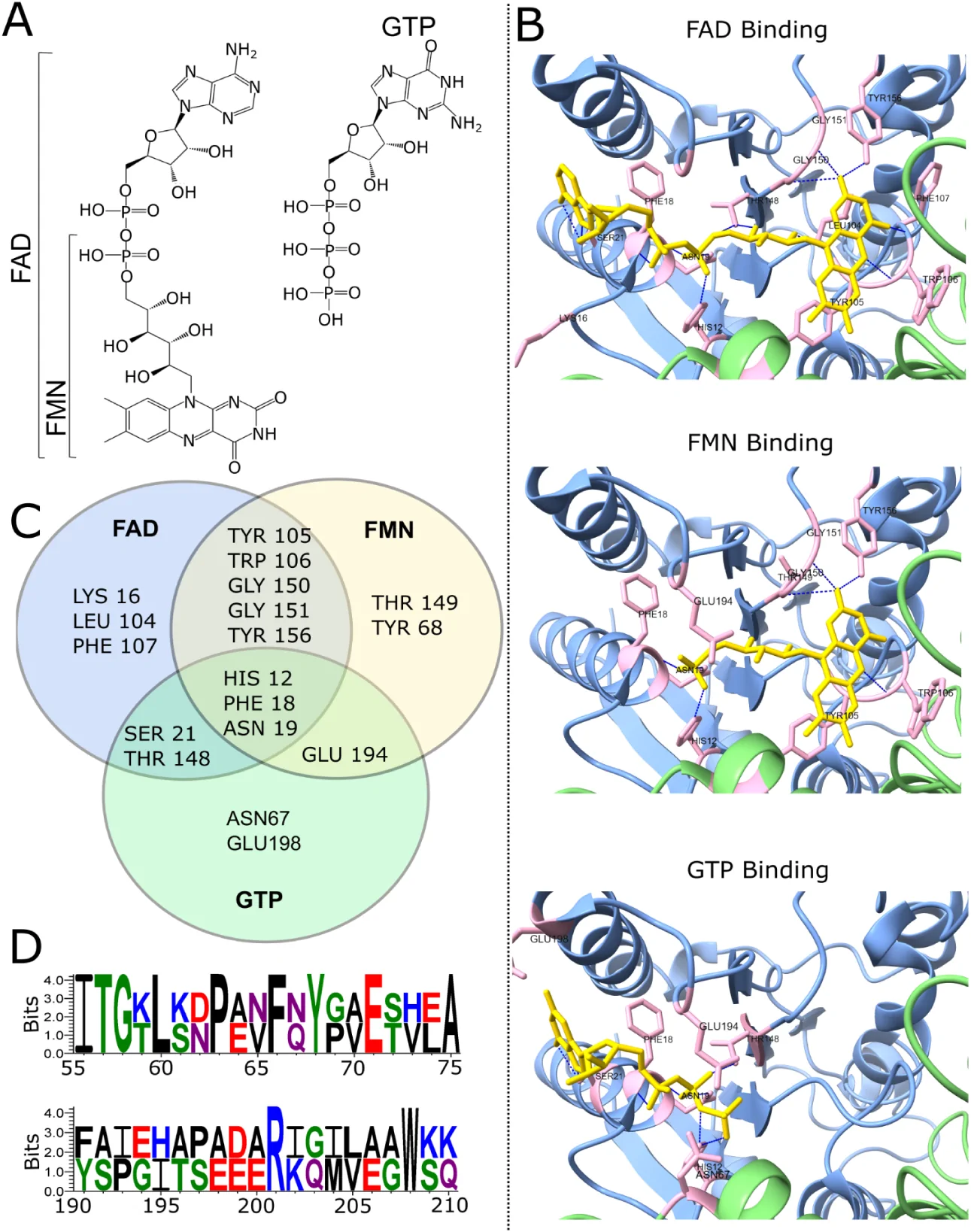
Structural comparison of FAD, FMN and GTP interactions with QR2. (A) Chemical structures of FMN, FAD, and GTP. (B) ChimeraX^36^ representations of QR2 interactions with FAD (top), FMN (middle), and GTP (bottom). FAD and FMN are shown from crystal structures 7O4D and 4QOF, respectively, whereas GTP was modeled using AlphaFold.^28^ Residues identified by both LigPlot^26^ and PLIP^27^ are shown in pink, ligands in yellow, and hydrogen bonds as dashed blue lines. Chain A and chain B are shown in green and blue, respectively, with all structures displayed in the same reference frame. (C) Venn diagram summarizing QR2 residues involved in FAD, FMN, and GTP binding, based on interactions identified by both LigPlot and PLIP, highlighting shared and ligand-specific contacts. (D) Sequence alignment of QR1 and QR2 across two 20-residue regions (positions 55–75 and 190–210) selected for their contribution to GTP-specific binding, generated using WebLogo.^37^ Colors denote amino-acid chemical properties: acidic (red), basic (blue), polar (green), neutral (purple), and hydrophobic (black).

QR2 interactions with FAD and FMN were analyzed using the PDB crystal structures 7O4D and 4QOF, respectively, whereas GTP interactions were analyzed using an AlphaFold-predicted^28^ QR2 model. LigPlot^26^ and PLIP^27^ were used to identify protein–ligand contacts. Structural comparison revealed that all three ligands occupy a highly overlapping region of the QR2 binding pocket, but engage partially distinct interaction networks (Figure 2B). Venn analysis further resolves these shared and ligand-specific interaction networks (Figure 2C). Specifically, FAD engages the largest interaction network, with 13 residues identified compared with 11 for FMN and 8 for GTP, consistent with its larger molecular architecture. FAD and FMN show the greatest similarity, sharing 8 of the 11 residues identified for FMN, including His12, Phe18, Asn19, Tyr105, Trp106, Gly150, Gly151, and Tyr156. GTP shares five contacts with FAD and four with FMN, including His12, Phe18 and Asn19, which were common to all three ligands. Each ligand also retains distinct peripheral contacts, comprising three FAD-specific, two FMN-specific and two GTP-specific residues. These differ in chemical character: FAD-specific contacts include charged, hydrophobic and aromatic residues, FMN-specific contacts are predominantly polar and aromatic, whereas GTP-specific residues are polar or charged. The extensive FAD-FMN overlap is also consistent with their shared planar flavin moiety, which is accommodated by aromatic and hydrophobic contacts involving Trp, Tyr and Phe residues that account for half of the shared interaction set. Notably, the conformation of this flavin moiety is itself sensitive to the chemical state. Reduction of FAD to FADH_2_ has been shown to induce bending of the isoalloxazine ring together with local and global structural changes in QR2^14^, underscoring the sensitivity of the protein to subtle changes in ligand chemistry. Hence, FAD and FMN engage a common flavin-binding framework, but the broader interaction network of FAD and the chemical sensitivity of the flavin moiety indicate that even closely related ligands can impose distinct structural constraints on QR2.

Spatial comparison showed that the flavin cofactors extend closer toward the dimer interface, whereas GTP occupies a more distal region of the binding pocket (Figure 2B; Supplementary Figure S2). Despite this difference, the three ligands retain a conserved interaction region involving His12, Phe18 and Asn19. In particular, the phosphate group occupying the position equivalent to the β-phosphate of GTP is spatially conserved across FAD, FMN and GTP. Although GTP contains multiple phosphate groups, this specific phosphate occupies the same region of the binding pocket as the phosphate moiety present in FAD and FMN. Oxygens from this conserved phosphate group form hydrogen bonds with both His12 and Asn19 across all three ligand complexes. This conserved phosphate-associated interaction may therefore provide a common anchoring element within an otherwise adaptable binding environment. Predicted ATP binding similarly occupies this nucleotide-binding region and retains the corresponding phosphate-containing moiety (Supplementary Figure S2). Overall, the ligand overlays reveal a conserved interaction core, centered on the phosphate-associated contacts with His12 and Asn19, while FAD and FMN extend further toward the dimer interface than GTP, suggesting a specific structural route by which ligand architecture may differentially influence QR2 dimer stabilization.

Beyond this conserved interaction core, the majority of residues contacting FAD, FMN and GTP are located within nonstructured regions of QR2, whereas only Phe18, Asn19 and Ser21 lie within defined secondary-structured elements. Of these, Phe18 and Asn19 are shared by all three ligands, suggesting this small, structured interaction core is surrounded by a more flexible ligand-binding environment. This arrangement may allow QR2 to retain conserved anchoring interactions while accommodating chemically distinct ligands through alternative peripheral contacts. The potential structural importance of such local changes is supported by the naturally occurring Phe47Leu polymorphism. Megarity et al.^13^ showed that the Leu47 variant is less stable toward thermal denaturation and proteolysis and displays greater conformational heterogeneity than the Phe47 variant, despite differing by only a single amino acid substitution. Together, these observations support a model in which a limited structured core provides conserved ligand recognition, while the surrounding less-structured regions accommodate ligand-specific interactions that may influence QR2 stability and conformation behavior.

Next, we compared QR1 and QR2 across two 20-residue regions containing the amino acids identified as unique to GTP binding (Figure 2D). Although several residue positions are conserved, both regions differ in their local sequence composition and chemical character. The 55–75 region shows partial similarity between QR1 and QR2, whereas the 190–210 region is more strongly divergent. Notably, position 198 is Ala in QR1 but Glu in QR2. The introduction of a negatively charged side chain at this position may alter the local electrostatic environment and contribute to the greater compatibility of QR2 with GTP or other nucleotide-like ligands relative to QR1. These differences may contribute to the distinct GTP-binding environment of QR2 and, more broadly, reshape the ligand-proximal region despite conservation of the overall fold and dimer architecture.

Collectively, these findings define a QR2 binding architecture comprising a conserved interaction core, centered on the phosphate-associated contacts with His12 and Asn19, surrounded by ligand-specific peripheral interactions. Differences in chemistry of these contacts and in the spatial extension of FAD, FMN and GTP toward the dimer interface provide a structural basis through which distinct ligands may differentially influence QR2 dimer stabilization and monomer–dimer equilibrium.

### 3.3 Ligand-Dependent QR2 Dimer Assembly

Based on our *in silico* analysis and nMS findings, we tested the effects of nucleotide (GTP) and flavins (FAD and FMN) on QR2 assembly by titrating ligands and monitoring changes in dimer abundance using nMS. The addition of GTP induced a shift toward QR2 dimer formation (Figure 3A,B), revealing a previously unreported nucleotide sensitivity of QR2 assembly. Rather than collapsing the population into a single dimer, GTP promoted redistribution among existing dimer-associated species, consistent with a graded and tunable stabilization mechanism. The observed mass increment is formally consistent with the binding of two FAD molecules or three GTP molecules. However, since no exogenous FAD was supplied, the GTP-dependent redistribution is more consistent with nucleotide incorporation and displacement of pre associated flavin.

**Figure 3:**
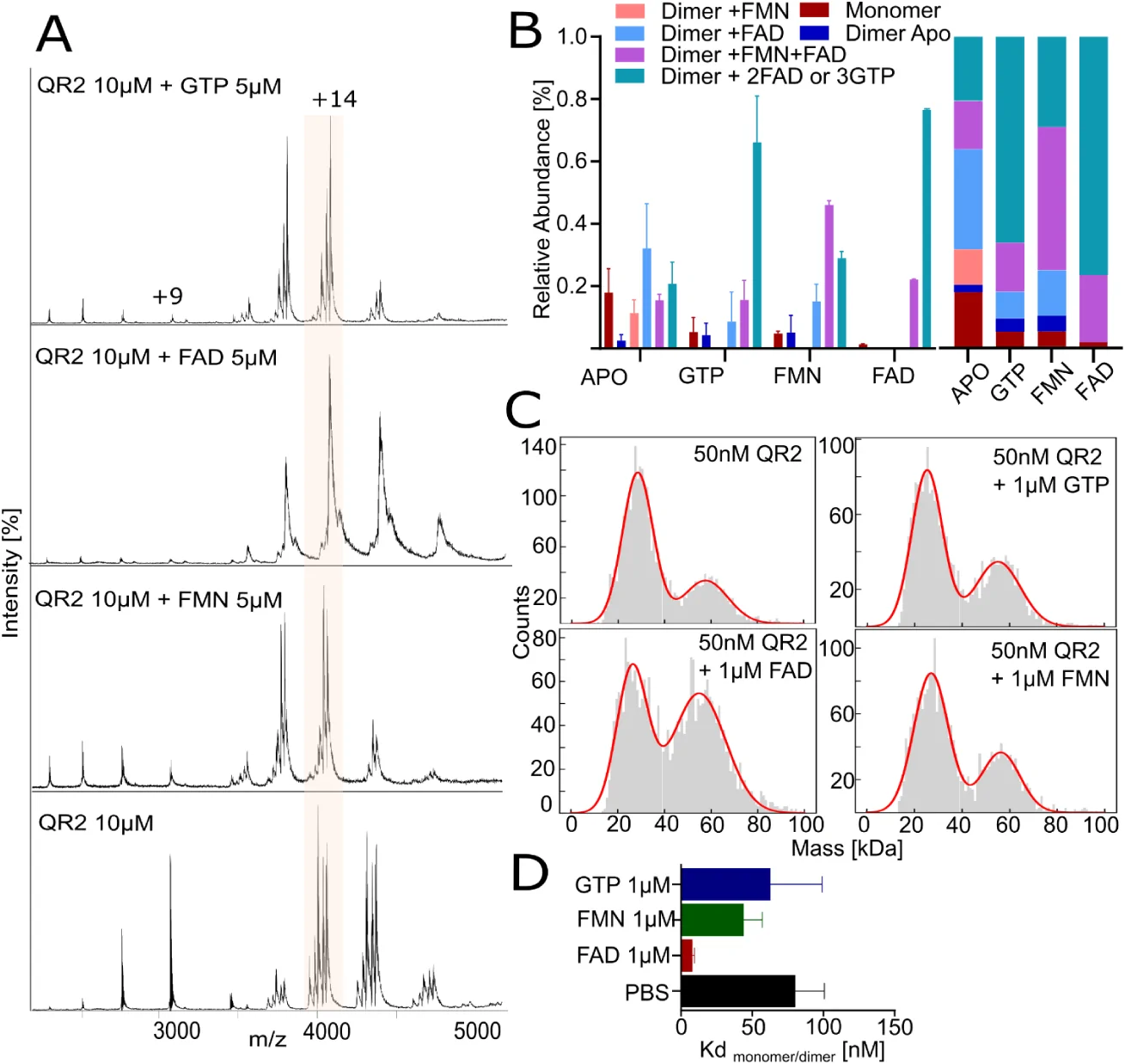
Ligand-dependent remodeling of QR2 oligomeric states measured by native mass spectrometry and mass photometry. (A) Native mass spectra of QR2 (10 μM) following incubation with 5 μM of GTP, FAD, and FMN. Each ligand induces redistribution of QR2 dimer-associated species relative to the protein without added ligand. (B) Quantification of relative abundances of monomeric and dimeric QR2 species under each condition. Bar plots show mean ± SD across replicates presented as relative abundance and as stacked bar charts to illustrate redistribution. (C) Mass photometry measurements of QR2 (50 nM) showing the oligomeric distribution in the absence of added ligand and following addition of 1 μM FAD, FMN, or GTP. Monomeric and dimeric populations are centered at approximately 28 and 56 kDa, respectively. (D) Monomer–dimer dissociation constants (*K*_d_)^30^ for QR2 alone (black) and in the presence of 1 μM FAD (red), FMN (green), or GTP (blue).

To assess whether three-GTP stoichiometry is structurally plausible, GTP molecules were modeled within the QR2 crystal structure PDB 7O4D (Supplementary Figure S2). The modeling showed that the volume occupied by a single FAD can accommodate two GTP molecules, providing sufficient space for up to four GTP molecules per QR2 dimer. Thus, experimentally inferred three-GTP occupancy is sterically compatible with the available binding volume. Although such stoichiometry is noncanonical, the observation of odd-numbered endogenous adducts in the native spectra further suggests that QR2 can accommodate asymmetric ligand occupancy.

This interpretation is also compatible with the intracellular metabolite environment of *E. coli*, where GTP concentrations have been reported at approximately 1.1–4.9 mM, substantially exceeding those of FAD (∼50–200 μM) and FMN (∼3–50 μM), giving an approximate abundance hierarchy of GTP ≫ FAD ≳ FMN. Together, the structural modeling, asymmetric nMS species and high intracellular concentration of GTP support the plausibility of multiple nucleotide occupancy within the QR2 dimer. Importantly, GTP binding also causes a shift in the *m*/*z* distribution of the dimer relative to native QR2, without a corresponding net mass change. This suggests that nucleotide binding may alter the conformational state of the dimer rather than simply exchange one bound cofactor for another.

Consistent with previous biochemical and structural studies, the addition of FAD produced a pronounced shift toward a dominant dimeric species (Figure 3A,B), confirming its role as a primary stabilizing prosthetic group. The binding of FAD effectively displaced alternative dimeric populations, yielding a highly uniform assembly and serving as an internal validation of the nMS approach. In contrast, FMN induced a markedly different response (Figure 3A,B and Supplementary Figure S3). FMN promoted dimer formation but simultaneously drove extensive remodeling of cofactor occupancy, characterized by enrichment of FMN-associated dimers and reciprocal depletion of FAD-bound species. This competitive exchange demonstrates that FMN can partially replace preassociated FAD, generating a heterogeneous ensemble of dynamically interconverting dimeric states. Unlike FAD, which enforces a rigid dimer, FMN engages QR2 more transiently, fine-tuning assembly rather than locking it into a single configuration. This reveals previously unknown functional aspects in the relationship between QR2 and FMN.

To independently validate the oligomeric behavior observed by nMS, QR2 was analyzed using mass photometry, an orthogonal technique that directly reports protein stoichiometry in solution.^29,38^ Measurements revealed a concentration-dependent monomer–dimer equilibrium, with an apparent dissociation constant (*K*_d_) of approximately 80 ± 20 nM for QR2 in the absence of added ligand. Addition of FAD, FMN or GTP shifted the population toward the dimeric state, although to different extents (Figure 3C). FAD produced the strongest effect, decreasing the apparent monomer–dimer *K*_d_ to 8 ± 1 nM, whereas FMN and GTP produced more moderate shifts, with *K*_d_ values of 44 ± 13 nM and 63 ± 36 nM, respectively (Figure 3D). These measurements quantitatively support the ligand-dependent redistribution observed by nMS, identifying FAD as the strongest stabilizer of the QR2 dimer while revealing more modest effects for FMN and GTP. Together, the nMS and mass photometry data demonstrate that QR2 dimerization is ligand-sensitive and that chemically distinct ligands differentially tune the monomer–dimer equilibrium.

Overall, these results reveal a hierarchy of ligands in the QR2 assembly, in which GTP alters the assembly state in a nucleotide-dependent manner, FAD imposes strong structural stabilization, and FMN enables dynamic cofactor exchange. In particular, FMN and GTP binding induce reproducible shifts in the QR2 dimer *m*/*z* envelope without corresponding changes in intact mass, consistent with ligand-driven conformational change that alters charge-state distribution. In contrast, FAD binding produces a single dominant dimer species without detectable *m*/*z* redistribution, indicative of a more rigid, structurally constrained assembly. This spectrum of responses highlights the pronounced structural heterogeneity in QR2 dimerization, demonstrating that chemically distinct ligands remodel QR2 into multiple coexisting dimeric conformers. Collectively, these findings establish QR2 as a ligand-responsive assembly whose quaternary structure is tuned by identity and availability of ligands, rather than protein concentration alone, revealing conformational heterogeneity as an integral characteristic of QR2 regulation.

### 3.4 Ligand-Dependent QR2 Dimerization Uncouples Oligomeric State from Catalytic Activity

To directly relate ligand-induced QR2 dimerization to enzymatic function, we compared oligomeric state and reductase activity in the presence of FMN, FAD, GTP, and QR2 inhibitor YB537 ^12^ under matched conditions. First, nMS of YB537 with QR2 showed that, similarly to flavins and GTP, results in the formation of QR2 dimers (Figure 4A,B and Supplementary Figure S4). However, in contrast to the distinct distribution of dimer-associated peaks and characteristic shifts along the *m*/*z* axis described for GTP and flavins, YB537 causes enrichment of the highest-mass dimer population. This species is shifted relative to the corresponding highest-*m*/*z* peak in native QR2, with its mass increase consistent with four sodiated YB537 molecules rather than the two FAD molecules assigned to the native state. This altered stoichiometry further supports ligand-dependent remodeling of the QR2 dimer and suggests that YB537 engages QR2 through a binding mode distinct from that of flavin cofactors, and provides new details regarding YB537 binding to QR2, beyond those revealed by the resolved crystal structure.^12^

**Figure 4:**
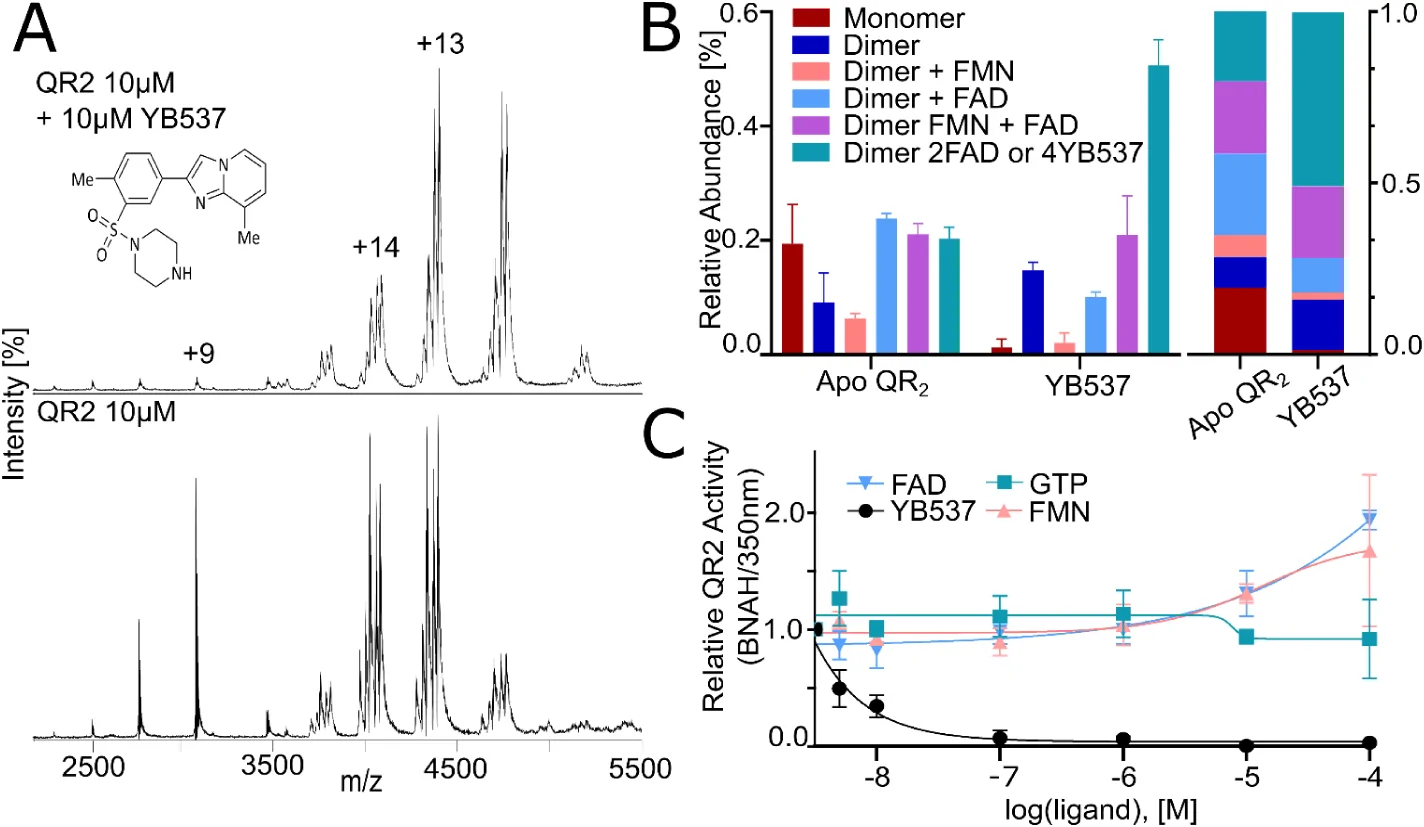
Modulation of QR2 dimer composition and reductase activity by YB537 and small-molecule ligands.

To assess whether these structurally distinct dimeric assemblies exhibit corresponding differences in catalytic competence, QR2 activity was measured using a spectrophotometric assay driven by the artificial hydride donor N-benzyldihydronicotinamide (BNAH)- and acceptor, menadione, and the reaction progress was monitored at 350 nm (Figure 4C).^12^ In the presence of flavin cofactors, both FAD- and FMN-bound QR2 display robust enzymatic activity. Although FAD produced the most homogeneous dimeric population as measured by nMS, FMN-supported dimers retain comparable catalytic output, indicating that variations in cofactor occupancy and dimer composition do not preclude reductase activity.

In contrast, the addition of GTP produces an attenuation of QR2 activity relative to flavin-bound states, consistent with nMS observations showing redistribution of cofactor-associated dimer species rather than complete displacement of catalytically competent flavins. As expected, YB537 prevents reductase activity, despite strongly stabilizing the dimeric assembly. This dissociation between dimer formation and catalytic output demonstrates that not all QR2 dimers are equivalent in their reductase activity.

Together, these results establish that QR2 dimerization alone is not predictive of catalytic activity. Instead, ligand identity dictates both the structural properties of the dimeric assembly and its functional competence. Flavins promote catalytically active dimers, GTP induces dimerization with reduced activity, and YB537 binding stabilizes inactive dimeric states. The observation that chemically distinct ligands universally enrich dimer formation but produce markedly different enzymatic profiles supports a model in which QR2 exists as a spectrum of ligand-defined dimer conformations, with functional output determined by small-molecule engagement rather than oligomeric state per se.

(A) Native mass spectra of QR2 (10 μM) following incubation with YB537 (10 μM) and chemical structure of the QR2 inhibitor YB537. (B) Quantitative analysis of dimer-associated species, presented as relative abundance and stacked bar charts to illustrate redistribution of cofactor-bound dimer populations upon inhibitor binding. (C) Functional reductase assays of QR2 (10 nM) in the presence of increasing concentrations of GTP, YB537, FAD, and FMN, showing ligand-dependent modulation of reductase activity.

## 4 Discussion and Conclusion

In this study, we show that human QR2 exists not as a single static holoenzyme, but as a ligand-responsive assembly whose quaternary structure is dynamically shaped by small-molecule engagement. Using nMS, we resolve multiple coexisting QR2 dimeric states stabilized by flavins, nucleotides, and the QR2 inhibitor YB537, revealing a previously unrecognized degree of structural plasticity in this enzyme. The QR2 monomer–dimer equilibrium is strongly influenced by ligand identity and availability, highlighting the chemical environment as an important determinant of QR2 assembly. This ligand dependence was corroborated by mass photometry, which showed a relative decrease of QR2 monomer–dimer *K*_d_ values. FAD produces the strongest shift toward the dimeric state, whereas FMN and GTP exert more moderate effects. Together with the multiple partially occupied dimers resolved by nMS, these measurements establish that QR2 does not populate a single dimeric state, but rather an ensemble of ligand-dependent assemblies whose relative abundance is shaped by the bound small molecules.

Our data further demonstrate that stable QR2 dimerization can occur upon binding of a single flavin cofactor, indicating asymmetric occupancy and structural adaptability. This contrasts with a simple model of symmetric dimer formation and suggests that QR2 can populate partially occupied, compositionally heterogeneous states. This behavior is consistent with a growing appreciation that dimeric enzymes can populate ensembles of ligand-defined conformations rather than discrete on–off states.^39^

Beyond flavins, we identify GTP as a previously unreported modulator of QR2 assembly. GTP induces dimerization while simultaneously remodeling the cofactor-bound population and shifting the *m*/*z* distribution without a corresponding change in intact mass, consistent with ligand-driven conformational rearrangement of the QR2 dimer. Although the precise nucleotide stoichiometry remains ambiguous, the observed redistribution strongly suggests displacement of endogenous flavins by GTP and highlights nucleotide binding as an additional regulatory axis for QR2. These findings expand the functional landscape of QR2 beyond redox cofactors and implicate the availability of cellular nucleotides as a potential determinant of the structural state of QR2.

Comparative *in silico* analysis further reveals that FAD and GTP engage QR2 through chemically distinct but largely overlapping interaction networks. Whereas FAD binding involves a broader constellation of residues, GTP engages a more restricted interaction network and substantially overlaps with contacts made by the adenosine monophosphate moiety of FAD. In particular, phosphate-associated oxygens at the position corresponding spatially to the β-phosphate of GTP form hydrogen bonds with both His12 and Asn19 similarly to flavin ligand complexes, providing a common anchoring feature despite substantial differences in ligand architecture. From this, it follows that other forms of purine nucleotides (e.g., GDP, ATP, adenosine) may similarly interact with QR2.

These, and other potential interactions, should be considered in future investigations. Differences in local flavin, nucleotide and other metabolite availability, together with cellular redox state, could shift the balance between QR2 assembly states and resultant functional output. This is especially relevant given the wide spread of tissues, cell types and subcellular localizations in which QR2 has been detected,^8,40,41^ in health and disease,^42,43^ in which the small molecule milieu is likely to differ, and affect QR2 structure and function accordingly. Thus, the local metabolite profile may represent an additional layer of QR2 regulation, providing a potential explanation of how this widely expressed protein can participate in such diverse processes.^4,6,11,14,40,44–46^

Importantly, the ligand-induced dimerization of QR2 is partly uncoupled from catalytic competence. Although flavins, GTP and YB537 all stabilize dimeric QR2, only flavin-bound assemblies support robust reductase activity, whereas GTP partially attenuates activity and inhibitor binding abolishes it entirely. These findings establish that dimer formation alone is not predictive of enzymatic function. Rather, ligand identity defines the functional output of the QR2 dimer. This uncoupling suggests that QR2 dimers may serve broader regulatory or signaling roles beyond quinone reduction, potentially enabling QR2 to operate as a ligand-responsive scaffold or molecular sensor.^14^ Consistent with this possibility, a recent study identified FAD-bound QR2, but not the apo dimer, as a reader of histone H3 serotonylation,^40^ with QR2 playing a role in epigenetic regulation. Substitution of residues involved in H3 serotonylation recognition abolished this epigenetic interaction, without disrupting reductase activity,^40^ further supporting the importance of fine structural tuning within the QR2 dimer. This demonstrates that relatively subtle local changes can selectively alter one functional output while preserving another.

These observations also align with emerging evolutionary and biochemical evidence that QR2 differs fundamentally from its paralog QR1.^4,5^ This includes markedly reduced catalytic efficiency with physiological nicotinamide cofactors and evolutionary selective pressure toward diminished redox activity. Together, these findings raise the possibility that QR2 may function in cells not solely as a reductase but as a structurally adaptive protein whose biological roles are mediated through ligand-dependent assembly states. Future studies using human cells, to analyze QR2 interactions by nMS in different cellular compartments in physiological and disease contexts, would be needed to fully explore these possibilities.

More broadly, our work illustrates the power of nMS and mass photometry to resolve dynamic protein assemblies and uncover regulatory mechanisms that remain inaccessible to static structural methods. By preserving noncovalent interactions and directly reporting on ligand occupancy and oligomeric state, these methods enable dissection of assembly landscapes that underpin functional diversity.

In summary, we propose that QR2 exists in a range of ligand-defined dimeric conformations whose structural and functional properties are tuned by the chemical environment. Ligand identity shifts the monomer–dimer equilibrium to different extents, while dimer formation itself does not dictate catalytic output. This ligand-dependent plasticity provides a mechanistic basis for QR2 regulation and suggests new strategies for therapeutic intervention, in which targeting assembly, rather than active sites alone, may offer more effective modulation of QR2 biology.

## Supplementary Material



## Notes

Supplementary Figures S1–S4 depicting native top-down mass spectrometry analysis of QR2; structural comparison of FAD, FMN, GTP, and ATP binding to QR2; FMN titration by native mass spectrometry; YB537 and GTP titrations by native mass spectrometry (PDF)
