# Supplementary material for: Quinone Reductase 2 Dimerization is Dynamically Driven by Ligand Binding
Source: Biochemistry. 2026 Sep 24;65(21):3559–68. doi: 10.1021/acs.biochem.6c00461 (PMC13738342; doi:10.1021/acs.biochem.6c00461)
Supplement: Supplementary file 1 [file bi6c00461_si_001.pdf]

Supporting Information: Quinone Reductase 2 Dimerization is Dynamically Driven by Ligand Binding

Maya Miller*[1,2,3], Dan Loewenthal[2,3], Philipp Kukura[2,3], and Nathaniel Gould*[3,4]

[1]*Department of Physiology, Anatomy and Genetics, University of Oxford, OXJ 3PT, UK*
[2]*DepartmentofChemistry, University of Oxford, OXJ 3TA, UK*
[3]*Kavli Institute for Nanoscience Discovery, University of Oxford, OXJ 3QU, UK*
[4]*Department of Psychiatry, University of Oxford, OX3 7JX, UK*

*Correspondence to: maya.miller@dpag.ox.ac.uk and nathaniel.gould@psych.ox.ac.uk

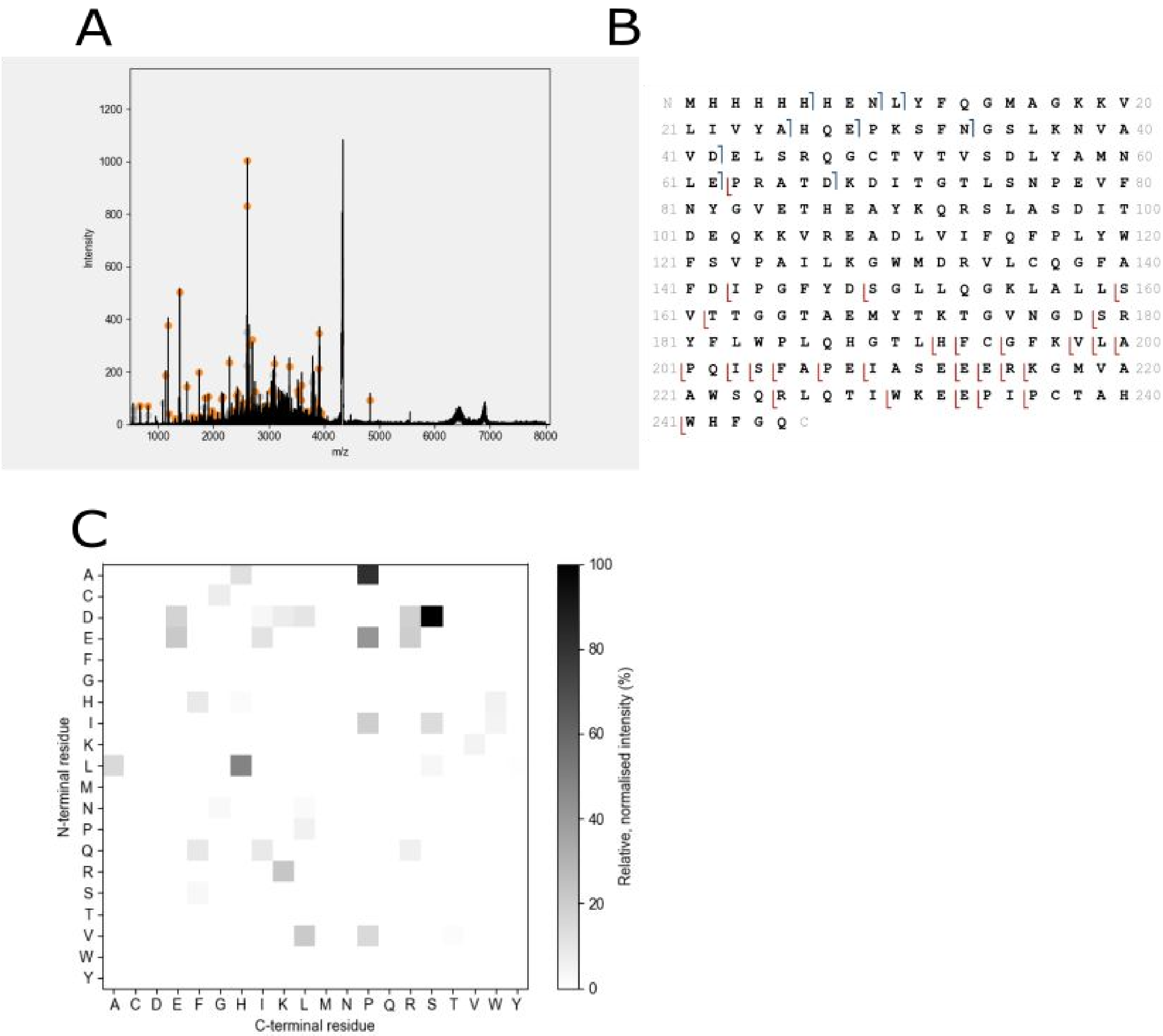

Figure S1. Native top-down mass spectrometry analysis of QR2 using PrecisION [1]. **(A)** Top-down mass spectrum chromatogram. Orange peaks represent deconvoluted ions, while gray peaks correspond to non-deconvoluted ions. **(B)** Sequence map coverage derived from top-down mass spectrometry, indicating identified fragment ions across the protein sequence. **(C)** PrecisION residue-pair heatmap for UniProt P16083 (+His-tag), illustrating high-confidence residue pair assignments in QR2.

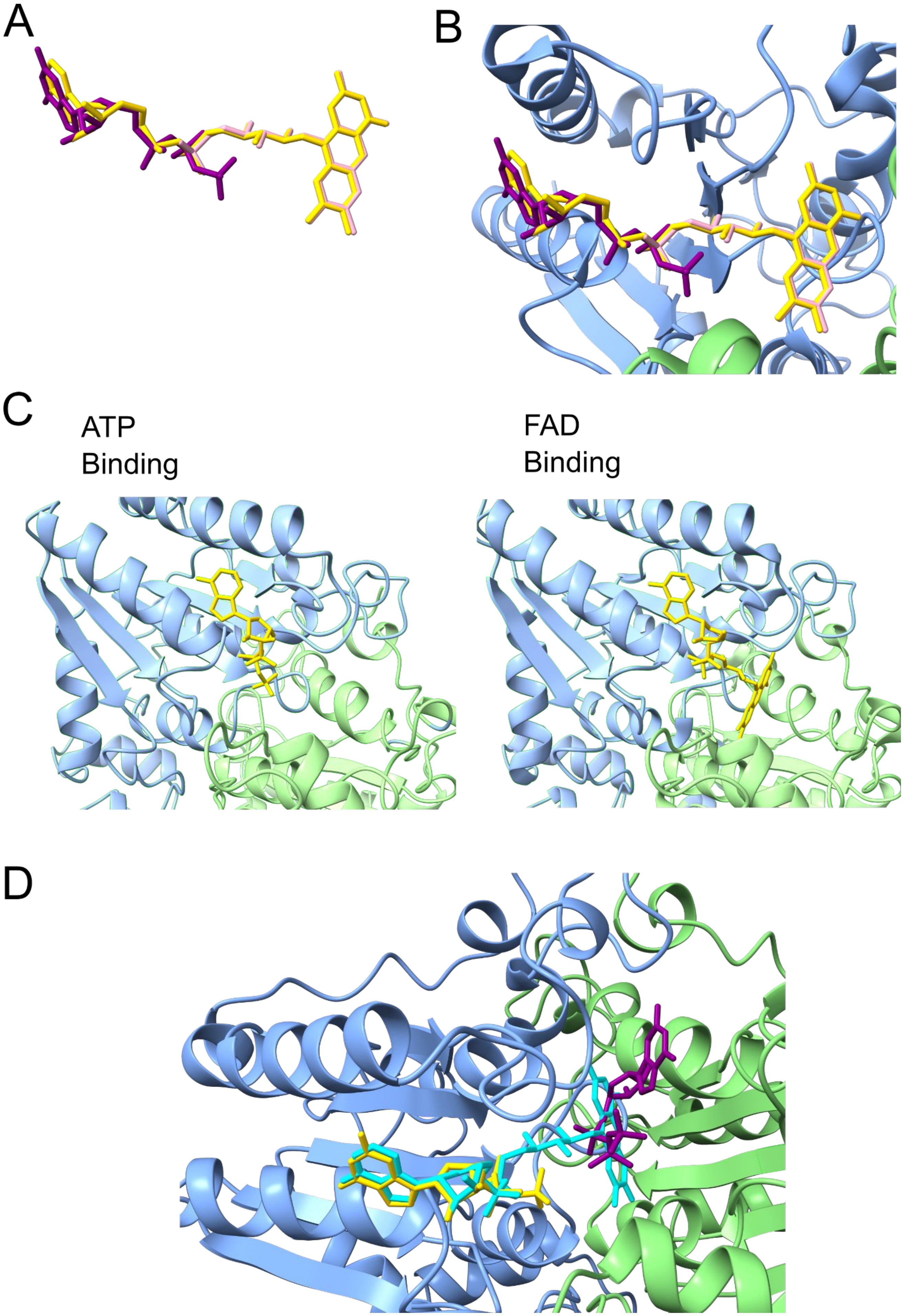
A
B
C
ATP
Binding
FAD
Binding
D

Figure S2. Structural comparison and ligand binding interaction of QR2. **(A)** Superposition of isolated FAD (yellow), FMN (pink), and GTP (purple) ligands in their aligned QR2 binding positions, shown without the surrounding protein. FAD and FMN positions derived from the crystal structures PDB 7O4D and 4QOF, respectively, whereas GTP was positioned using an AlphaFold-predicted[2] structural model. **(B)** FAD, FMN, and GTP positioned within the QR2 binding pocket to illustrate their relative locations and potential interactions with the protein. **(C)** QR2 interactions with FAD, based on the crystal structure PDB 7O4D, and with ATP, based on an AlphaFold-predicted structural model. QR2 is shown as a dimer, with chain A in green and chain B in blue; bound ligands are shown in gold. (**D)** Structural modelling of GTP occupancy within the QR2 FAD-binding region, based on PDB 7O4D, with FAD shown in cyan and two GTP molecules shown in yellow and purple. The two QR2 protomers are shown in green and blue, respectively.

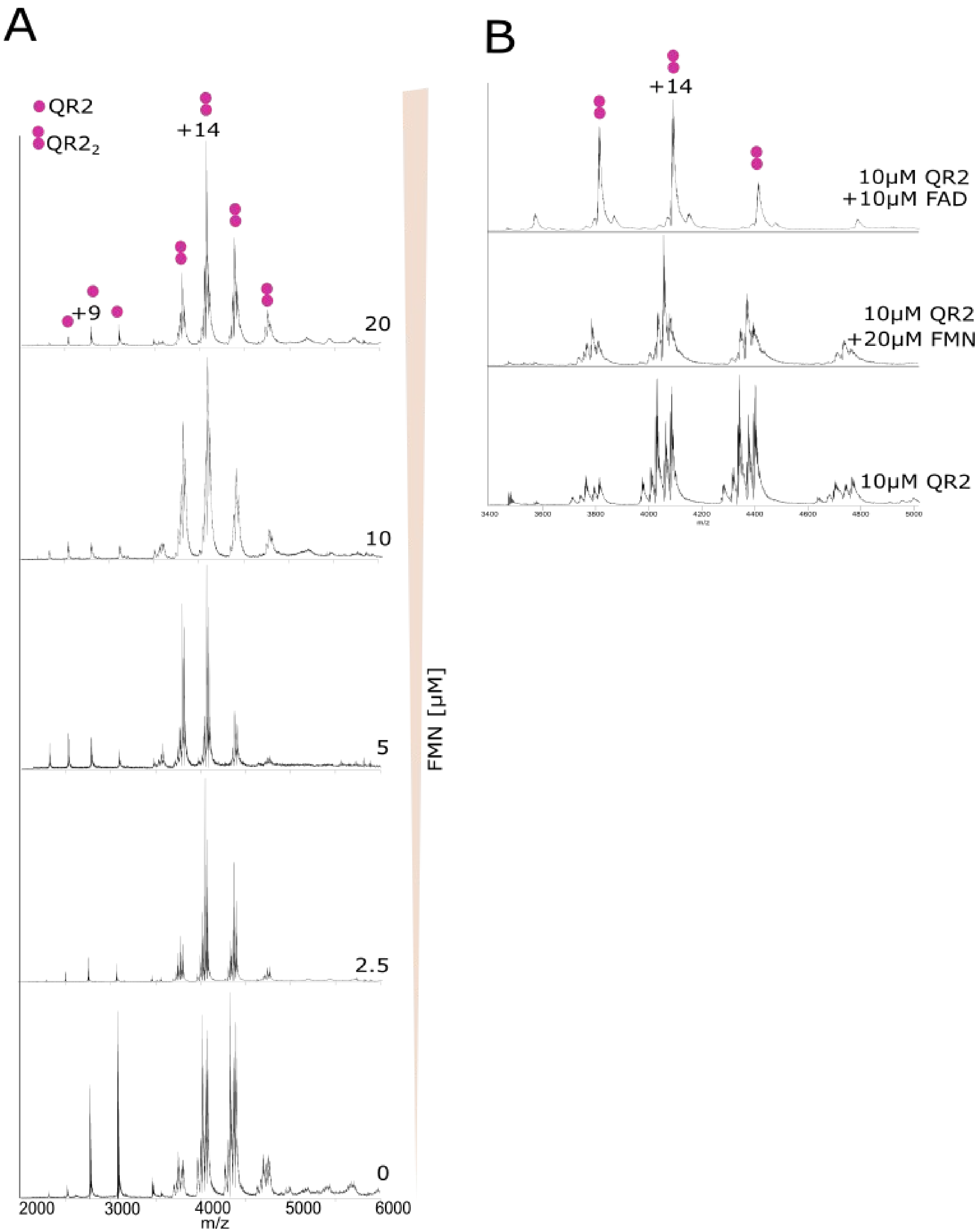

Figure S3. (A) Native mass spectrum of 10 µM QR2 spiked with increasing concentrations of FMN (0-20 µM). **(B)** Comparison of native mass spectra of QR2 alone, QR2 with 20 µM FMN, and QR2 with 10 µM FAD.

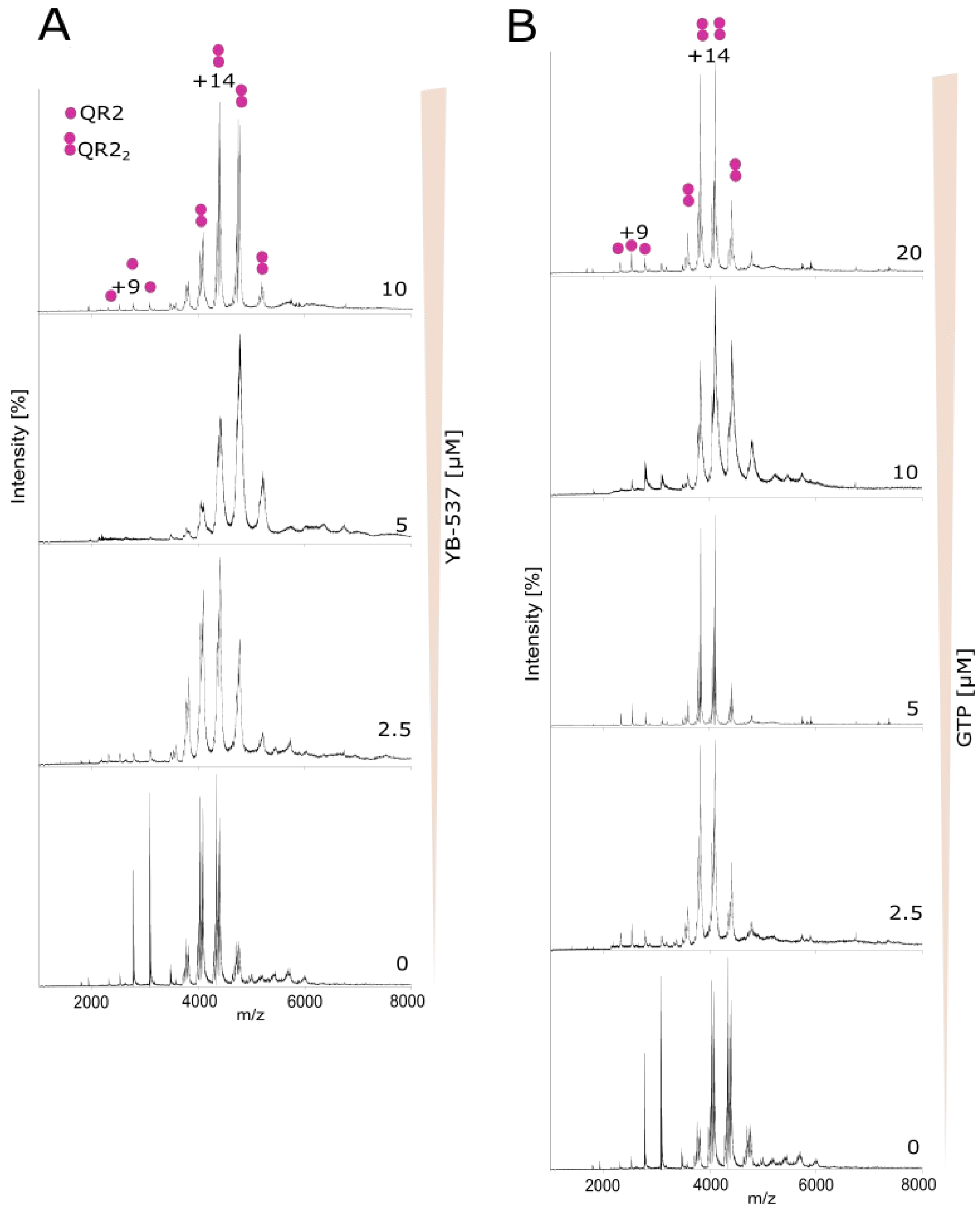

Figure S4. (A) Native mass spectrum of 10 µM QR2 spiked with increasing concentrations of inhibitor YB537 (0-10 µM). **(B)** Native mass spectrum of 10 µM QR2 spiked with increasing concentrations of GTP (0-20 µM).

References

(1) Bennett, J. L.; El-Baba, T. J.; Zouboulis, K. C.; Kirschbaum, C.; Song, H.; Butroid, F. I.; Benesch, J. L. P.; Lutomski, C. A.; Robinson, C. V. Uncovering Hidden Protein Modifications with Native Top-down Mass Spectrometry. *Nat. Methods* **2025**, *22* (10), 2127–2137. https://doi.org/10.1038/S41592-025-02846-5;SUBJMETA.

(2) Abramson, J.; Adler, J.; Dunger, J.; Evans, R.; Green, T.; Pritzel, A.; Ronneberger, O.; Willmore, L.; Ballard, A. J.; Bambrick, J.; Bodenstein, S. W.; Evans, D. A.; Hung, C. C.; O'Neill, M.; Reiman, D.; Tunyasuvunakool, K.; Wu, Z.; Žemgulytė, A.; Arvaniti, E.; Beattie, C.; Bertolli, O.; Bridgland, A.; Cherepanov, A.; Congreve, M.; Cowen-Rivers, A. I.; Cowie, A.; Figurnov, M.; Fuchs, F. B.; Gladman, H.; Jain, R.; Khan, Y. A.; Low, C. M. R.; Perlin, K.; Potapenko, A.; Savy, P.; Singh, S.; Stecula, A.; Thillaisundaram, A.; Tong, C.; Yakneen, S.; Zhong, E. D.; Zielinski, M.; Žídek, A.; Bapst, V.; Kohli, P.; Jaderberg, M.; Hassabis, D.; Jumper, J. M. Accurate Structure Prediction of Biomolecular Interactions with AlphaFold 3. *Nature 2024 630:8016* **2024**, *630* (8016), 493–500. https://doi.org/10.1038/s41586-024-07487-w.
